# Effect of Motion-Controlled Video Games-Based Virtual Reality Exercise on Patients with Post-COVID-19 Condition: A Randomized Controlled Trial

**DOI:** 10.3390/healthcare13222914

**Published:** 2025-11-14

**Authors:** Musa Polat, Pınar Oba, Ahmet Karadağ

**Affiliations:** 1 Department of Physical Medicine and Rehabilitation, Faculty of Medicine, Sivas Cumhuriyet University, Sivas 58140, Türkiye; 2 Health Services Practice and Research Hospital, Sivas Cumhuriyet University, Campus of Cumhuriyet University, Sivas 58140, Türkiye; 3 Clinical of Physical Medicine and Rehabilitation, Yozgat City Hospital, Yozgat 66100, Türkiye

**Keywords:** exercise, post-acute COVID-19 syndrome, rehabilitation, virtual reality

## Abstract

**Objective**: Virtual reality (VR) exercises may offer a comprehensive rehabilitation approach for many conditions. This study primarily aimed to evaluate the effectiveness of VR exercises compared with conventional exercise in reducing pain intensity in individuals with post-COVID-19 condition (PCC). Secondary analyses explored their effects on fatigue, functional capacity, mood, and quality of life. **Materials and Methods**: A single-center, randomized, assessor-blinded intervention study was conducted with 79 individuals between July 2021 and February 2022. The primary outcome was pain intensity measured using the Visual Analog Scale (VAS). Secondary outcomes included mood (Hospital Anxiety and Depression Scale, HADS), fatigue (Fatigue Severity Scale, FSS), quality of life (SF-12), and functional exercise capacity (6-Minute Walk Test, 6 MWT). Participants completed supervised exercise sessions 3 times weekly for 30–45 min over 8 weeks. The conventional exercise program involved moderate-intensity aerobic, strength, stretching, and neuromuscular exercises. VR exercises were delivered semi-immersively using motion-controlled video games. Time × group interactions were analyzed using linear mixed-effects model. **Results**: In both groups, 6MWT, SF-12 physical and mental components increased, while VAS, FSS and HADS anxiety and depression scores decreased. Time-group interaction was observed in favor of VRG for VAS [*F*(1, 59.4) = 56.3, *p* = 0.001], as well as HADS-D [*F*(1, 54.6) = 7.40, *p* = 0.008] and FSS [*F*(1, 61.4) = 8.96, *p* = 0.004]. **Conclusions**: While structured exercise improves the physical and psychological conditions of individuals with PCC, virtual reality exercises stand out in pain, also fatigue, and depression.

## 1. Introduction

While the vast majority of recovered patients infected with SARS-CoV-2 have no remaining symptoms, approximately 10% of individuals developed post-COVID-19 condition (PCC) [1]. PCC is characterized by symptoms such as fatigue, shortness of breath, muscle or joint pain, and mood disorders related to one or more body systems and is associated with medium to long-term effects [2]. The persistence of the SARS-CoV-2 virus, altered immune responses, autoimmunity, and microthromboses in different body systems are implicated in the etiology of PCC [3]. Although symptoms are expected to improve within 4–9 months, approximately 15% of people with PCC still experience symptoms after 12 months [4]. Therefore, proper management of PCC prevents individual, social, and economic problems. In clinical practice, personalized treatments tailored to a patient’s individual needs and the physician’s experience are used for symptomatic relief; however, there is limited research on this topic.

Research data suggests that holistic care may help patients regain their functions and improve their quality of life [5]. Considering the contribution of virtual-reality (VR) exercises to both somatic and psychological recovery processes in different clinical groups, integrating VR exercises into the rehabilitation of patients with PCC may provide a holistic rehabilitation opportunity [6].

Reports from a variety of conditions, including chronic pain, multiple sclerosis, stroke, cardiopulmonary system diseases, mood disorders, and psychosomatic diseases, indicate that VR exercises can help to reduce pain intensity and fatigue, enhance motor abilities, improve quality of life, boost mood by mitigating symptoms of anxiety and depression, increase cognitive functions, and provide anxiolytic effects [7,8,9,10,11,12,13,14]. Although these diseases have different pathologies than PCC, many treatment goals and obstacles encountered are similar.

The therapeutic potential of motion-controlled, video game–based VR under supervised clinical conditions may stem from its unique integration of physical activation and cognitive engagement. The immersive and interactive environment facilitates attentional distraction from pain, while graded task difficulty promotes safe, progressive activation of the neuromuscular system without overexertion—an essential consideration for fatigue-prone PCC populations [6,7,8]. Concurrently, real-time feedback and goal-oriented challenges enhance motivation, emotional regulation, and self-efficacy, contributing to mood improvement through behavioral activation and positive reinforcement [9,10,11,12,13]. These mechanisms are consistent with previous findings demonstrating VR-induced modulation of pain, fatigue, and mood pathways across chronic pain, neurological, and cardiopulmonary rehabilitation contexts [7,8,9,10,11,12,13,14]. When delivered under professional supervision, these mechanisms operate within a controlled therapeutic framework, ensuring both safety and individualized progression [6,14].

In a limited number of previous studies, VR-assisted rehabilitation has been applied to individuals with PCC, either in hospital or home settings [15,16,17,18]. Evidence suggests that VR-enhanced exercises increase the 6 min walk distance, reduce anxiety, and lower dyspnea scores, as well as reduce fatigue, with effects comparable to those of conventional treatments [19]. The methodologies of these studies emphasize the concentration and treatment compliance-enhancing properties and cognitive contributions of VR applications, but motion-controlled video game-based VR exercises with a sensorimotor component are not included. This study aimed to investigate the effectiveness of motion-controlled video game–based VR exercises in improving pain intensity as the primary outcome, as well as their effects on fatigue, mood, functional capacity, and quality of life in individuals with PCC. A secondary aim was to compare these effects with those of conventional exercise.

## 2. Method

### 2.1. Study Design, Setting, and Ethical Considerations

This study is a single-center, randomized, evaluator-blind intervention study conducted at a tertiary university hospital between July 2021 and February 2022. The research protocol was approved by the local ethics committee (date: 23 June 2021, decision no: 2021-06/32), and written and verbal consent was obtained from all participants. The study was conducted in accordance with the Helsinki Declaration, Good Clinical Practice guidelines and the CONSORT 2025 recommendations. This trial was registered at the ClinicalTrials.gov (ID number NCT04983394, Date of registration: 28 July 2021).

### 2.2. Participants

79 individuals aged 18–65 years who were diagnosed with COVID-19 with a PCR test result in an oropharyngeal or nasopharyngeal swab sample taken more than 90 days ago and who had PCC symptoms for at least eight weeks were included. Patients with PCC who had been hospitalized, had signs of pneumonia or organ failure, had suffered an acute myocardial infarction or orthopedic surgery within the last 2 years, had cardiovascular system diseases such as uncontrolled hypertension, arrhythmia, or cardiac insufficiency, had known chronic respiratory system diseases, those with conditions causing widespread pain such as fibromyalgia syndrome, those with primary any neurological system diseases, or those unable to mobilize for any reason were excluded from the study.

### 2.3. Evaluation

The same physiatrist (eight years of experience) systematically evaluated the sociodemographic data, medical history including the time elapsed since the diagnosis of COVID-19, the duration of PCC symptoms, and outcome measures (fatigue level, pain intensity, general health status, emotional state, functional capacity) through face-to-face interviews at the hospital, and recorded them in a standardized form.

Outcome measures were reassessed after intervention by the same physiatrist who remained blinded to group allocation throughout the study. To ensure evaluator blinding, the physiatrist was not involved in the randomization or treatment procedures and did not have access to the allocation list. Participants were instructed not to disclose their group assignment or intervention type during evaluations. The physical therapist who administered the exercises worked in a separate room and did not communicate participants’ group identities to the evaluator. The 1st and 2nd evaluations were recorded on different forms, and the physiatrist had no knowledge of the initial evaluation scores.

### 2.4. Outcome Measures

#### 2.4.1. Primary Outcome

Pain intensity was assessed using the visual analog scale (VAS). The VAS is a one-dimensional pain scale used to measure and monitor pain intensity. Scores are rated from 0 points for “No pain” to 10 points for “The worst pain imaginable”. Participants were asked to mark the intensity of their pain over the past week on a 10 cm line.

#### 2.4.2. Secondary Outcomes

The Hospital Anxiety and Depression Scale (HADS) was used to assess participants’ mood states. This self-report scale consists of a total of 14 questions, including seven anxiety items and seven depression items. Each item is scored between 0 and 3 points, with higher scores indicating more severe anxiety or depressive symptoms [20].

The Fatigue Severity Scale (FSS) consists of nine items that measure the severity of fatigue symptoms experienced by participants over the past week. Each item is scored from 1 to 7. A score of “1” indicates strong disagreement, while a score of “7” indicates strong agreement [21].

Participants’ health-related quality of life was assessed using the Short Form-12 (SF-12), which assesses participants’ physical and mental health in 12 questions. Scores are calculated in two main dimensions: the physical component summary and the mental component summary. Lower scores indicate poorer health-related quality of life [22].

Participants’ functional exercise capacity was assessed using the 6-Minute Walk Test (6MWT). The test, administered according to the standard protocol, is based on the individual walking as fast as possible for 6 min along a 30 m straight corridor. During the test, participants were reminded not to run, that they could slow down or pause briefly if necessary, but that they must complete the test. At the end of the time period, the distance walked by the participant was recorded in meters [23].

### 2.5. Randomization

A physiatrist (three years of experience) who was not involved in the evaluation randomized participants to either the VR group (VRG) or the conventional exercise group (CTG) using a random number sequence generated by the Random Number Generator function in IBM SPSS Statistics v22 (IBM Corp., Armonk, NY, USA) with a 1:1 allocation ratio. The document indicating the group was placed in an opaque, sealed envelope and directed to the physical therapist who would perform the treatment procedures.

### 2.6. Intervention

Both intervention protocols were standardized in structure but individually adjusted in intensity and progression. Exercise sessions were conducted under the supervision of a physical therapist (seven years of experience) at the rehabilitation clinic for 30–45 min, three days per week, for eight weeks. In both groups, exercise difficulty was progressively increased according to each participant’s performance and tolerance.

#### 2.6.1. CTG

The conventional exercise program included moderate-intensity aerobic, strength, stretching, and neuromuscular exercises, based on the FITT-VP exercise prescription principles established by the American College of Sports Medicine and the recommendations outlined in Stanford Hall’s consensus statement on PCC rehabilitation [24,25].

Flexibility exercises were performed during the warm-up and cool-down periods of aerobic exercise for 3–5 min, consisting of static and dynamic upper and lower extremity stretching exercises in standing and sitting positions. Neuromuscular exercises were performed on the Bosu ball and balance board with eyes open and closed, using one or both feet.

Aerobic exercise using a bicycle ergometer was performed for 20–30 min, depending on patient tolerance, starting at 60% of the age-determined maximum heart rate and increasing by 5% every 2 weeks (75% in the last 2 weeks).

Strengthening exercises were performed for 10–15 min using dumbbells or sandbags targeting the large muscles of the upper and lower extremities. Initially, one set of strengthening exercises was performed with 8 repetitions targeting 6 different muscles at 50% of 1 RM intensity. Depending on patient tolerance, the exercise intensity was increased weekly to 70%, the number of muscles worked to 10, the number of sets to 2, and the number of repetitions per set to 12.

#### 2.6.2. VRG

VR exercises were implemented semi-immersively in the Kinect Sports Rivals program via the Microsoft Xbox One Kinect game console and Kinect V2 infrared camera. Participants were positioned 2 m away from a 55-inch LCD screen in a well-lit room. Motion-controlled video games, including tennis, bowling, soccer, target shooting, climbing, and jet ski racing, were played against a virtual opponent. Participants performed plyometric movements such as jumping and shooting, as well as limb and spine movements, to direct the avatar and ensured the coordination of their movements.

The games had multiple difficulty levels. The first session started at a basic level and progressed weekly according to each participant’s ability to complete tasks and defeat the virtual opponent.

All VR sessions were conducted under the direct supervision of a physiotherapist, who managed game transitions and difficulty adjustments. The physiotherapist also ensured correct posture, safety, and adherence to task requirements, while providing verbal encouragement and monitoring.

During the 8-week intervention, participants were instructed not to engage in any additional structured exercise, physiotherapy, or psychological treatment programs. Daily routines were maintained, and no changes in medication or other concomitant care were permitted unless clinically necessary.

### 2.7. Adverse Events Monitoring

Participants were monitored throughout all sessions for potential adverse events, including dizziness, nausea, headache, visual discomfort, muscle or joint pain, and excessive fatigue, cardiovascular or autonomic symptoms such as palpitations, shortness of breath, or lightheadedness by the physiotherapist. Post-exertional malaise (PEM) was specifically monitored by questioning participants before and after each session about disproportionate fatigue, muscle soreness, or prolonged recovery.

### 2.8. Statistical Analysis

The sample size was calculated based on the effect size derived from a preliminary study involving 20 participants. A two-tailed *t*-test was conducted using the G*Power 3.1 program (Heinrich Heine Universität Düsseldorf, Germany) with 80% power and a two-sided α of 0.05 to compare change scores between groups. Among all outcome measures, the largest sample requirement was observed for the VAS, designated as the primary outcome, with an estimated effect size of *d* = 0.67. Accordingly, a minimum of 36 participants per group was required. Considering a 10% anticipated dropout rate, a total of 79 participants were recruited to ensure adequate statistical power.

Statistical analyses were performed per protocol using the SPSS v22 computer program (IBM Corp., Armonk, NY, USA), with a Type I error level of 0.05. Both analytical and visual methods were used to determine whether variables were normally distributed. For comparing the sociodemographic data and baseline outcome measures of the groups, the chi-square test was used for categorical variables (gender, comorbidity), and the independent groups *t*-test was used for quantitative variables because showed normal distribution. The effects of the interventions on outcome measures were analyzed using a linear mixed-effects model (LMM) with group (CTG vs. VRG) as a between-subject factor and time (baseline vs. week 8) as a within-subject factor. The time × group interaction term was examined to determine whether the change over time differed between the groups. The LMM approach was preferred because it provides robust estimates in the presence of missing data and does not require the sphericity assumption of repeated-measures ANOVA. To further interpret clinical relevance, individual change scores were dichotomized based on the minimal clinically important difference (MCID) for each primary and secondary outcome. The proportion of participants achieving MCID in each group was compared using risk difference, from which the number needed to treat (NNT) was calculated. To visualize the time × group interaction of the outcome measures, line graphs were combined with bar graphs created using the mean and standard error.

## 3. Results

**Figure 1:**
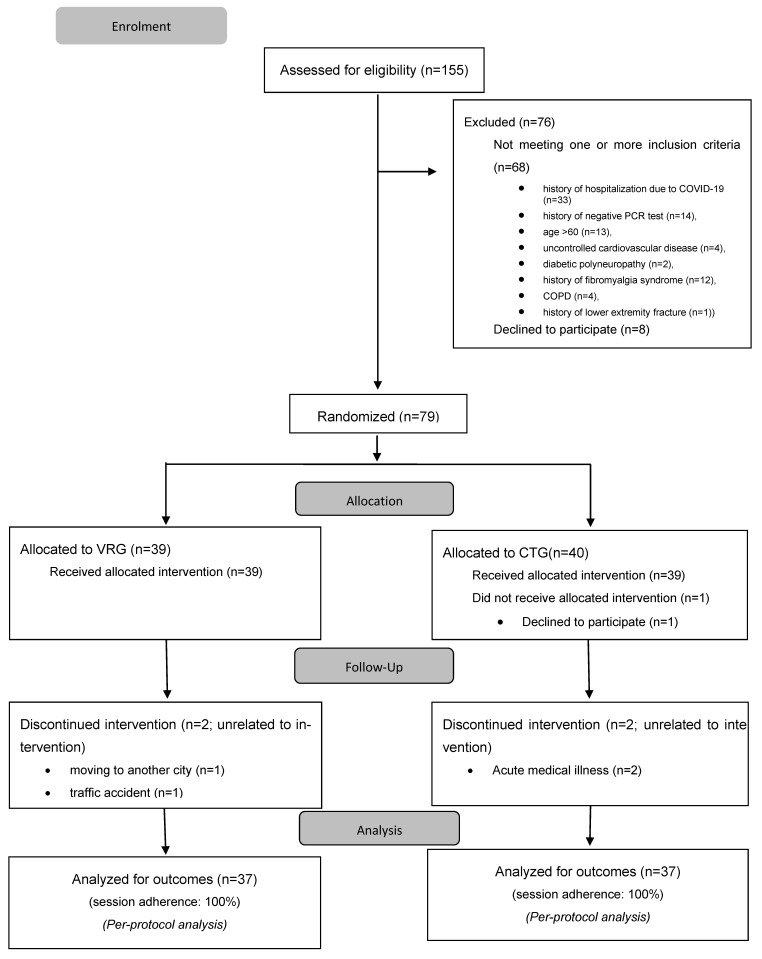
Study Flow Chart.

Among the 155 individuals evaluated, 68 did not meet one or more eligibility requirements and 8 declined to participate, leaving 79 participants who were randomized into two groups. Five participants withdrew from the study for reasons unrelated to the interventions. Thirty-seven participants in each group completed interventions (Figure 1).

The group’s sociodemographic characteristics were similar (Table 1). In the VRG, the time since COVID-19 diagnosis was 9.1 (2.2) months, and the PCC diagnosis period was 6.8 (1.3) months. In the CEG, the corresponding values were 8.7 (1.3) and 7.3 (1.7) months, respectively. No significant differences were observed between the groups (*p* = 0.67 and *p* = 0.42, respectively).

Baseline measurements were comparable between groups across all outcomes. After the 8-week intervention, 6MWT distance and SF-12 physical and SF-12 physical mental component scores increased, whereas VAS, FSS, HADS-A, and HADS-D scores decreased in both groups (Table 2 and Table 3). A ≥30% reduction (minimally important difference, MID) in pain intensity was achieved by 76% of participants in the VR group and 54% in the control group, corresponding to a number needed to treat (NNT) of 4.5. The proportion of participants achieving at least a 10% reduction in FSS was 68% in VRG and 49% in CTG (NNT ≈ 4). Approximately 61% of VRG participants achieved a clinically relevant improvement (≥1.5 points) in HADS-D compared with 42% in CTG (NNT = 6). The 6MWT exceeded the established MID of +30 m in 57% of VRG and 48% of CTG participants. Clinically meaningful improvement was observed in 62% vs. 57% of participants for HADS-A (≥2 points), 68% vs. 70% for SF-12 Physical (≥3 points), and 65% vs. 72% for SF-12 Mental (≥3 points), in VRG and CTG, respectively.

**Figure 2:**
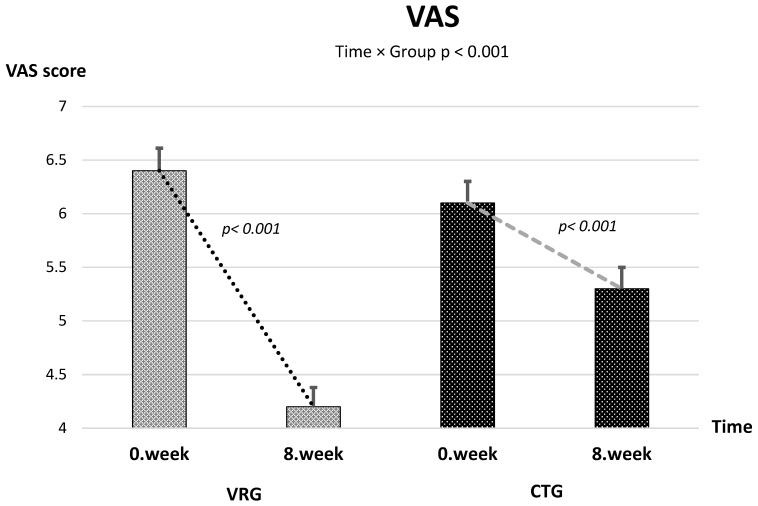
Change in VAS Scores Over Time Across Groups.

**Figure 3:**
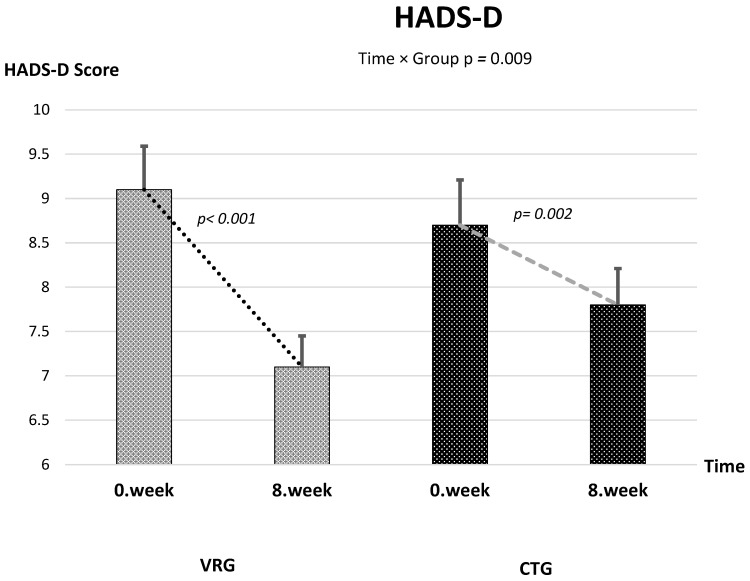
Change in HADS-D Scores Over Time Across Groups.

**Figure 4:**
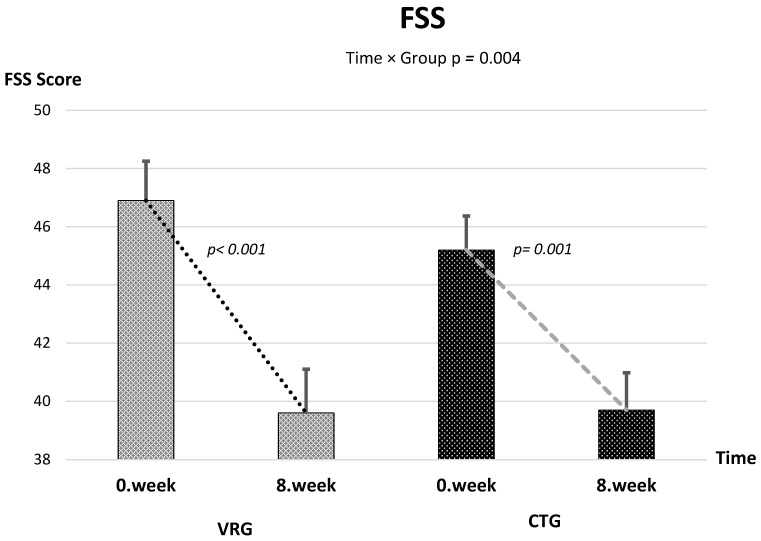
Change in FSS Scores Over Time Across Groups.

The linear mixed-effects model revealed a significant time × group interaction, indicating a greater reduction in pain in the VRG compared with the CTG [*F*(1, 59.4) = 56.31, *p* < 0.001] (Figure 2). The mean difference in change between groups was −1.37 (95% CI −1.73 to −1.00), corresponding to a large effect size (partial η^2^ = 0.487). Both groups showed significant within-group improvement over 8 weeks [*F*(1, 59.4) = 232.3, *p* < 0.001], with no baseline difference between them (*p* = 0.387). Also, time × group interaction was observed in favor of VRG for FSS (mean difference in change = −4.19, 95% CI −6.99 to −1.39; *F*(1, 61.49) = 8.96, *p* = 0.004; partial η^2^ = 0.127) and HADS-D (mean difference in change = −1.16, 95% CI −2.02 to −0.31; *F*(1, 54.64) = 7.40, *p* = 0.0087; partial η^2^ = 0.119) (Figure 3 and Figure 4). The time × group interaction was not statistically significant for 6MWT [*F*(1, 48.8) = 1.90, *p* = 0.175], HADS-A [*F*(1, 59.98) = 0.28, *p* = 0.601], SF-12 Physical [*F*(1, 69.54) = 0.009, *p* = 0.926] and SF-12 Mental [*F*(1, 71.4) = 0.76, *p* = 0.388] (Table 3).

No cases consistent with post-exertional symptom exacerbation were reported. In addition, no adverse events, injuries, or exercise intolerance were observed during or between sessions. Participants did not experience dizziness, nausea, or musculoskeletal discomfort requiring session interruption or medical attention.

## 4. Discussion

We compared the effectiveness of motion-controlled video game–based VR exercises with conventional exercise programs in patients with PCC. We observed greater improvements in pain and larger reductions in depressive symptoms and fatigue severity in the VRG. In addition, we observed that both interventions improved functional capacity and quality of life. To our knowledge, this is the first randomized controlled trial to directly investigate supervised, motion-controlled, video game–mediated VR exercises delivered in a hospital setting for individuals with PCC.

Early studies on PCC management, which viewed COVID-19 as primarily a respiratory disease, focused mainly on pulmonary rehabilitation [26]. It later became clear that PCC symptoms and their severity are not directly related to the initial infection [27]. Also, PCC have high heterogeneity of symptoms and underlying pathophysiological processes, as well as evidence that a psychosomatic component may also be present [1,3,28]. Consequently, rehabilitation approaches are now understood to require individualized, multidimensional strategies rather than focusing solely on pulmonary rehabilitation [29]. In this context, aerobic, resistance, flexibility, and mind–body exercises have all been implemented [30,31,32,33]. However, the fluctuating nature of PCC symptoms, the risk of PEM, and challenges with adherence have posed difficulties for exercise-based interventions [34].

It has been reported that structured, supervised exercise is generally beneficial in PCC, regardless of the exercise modality [5,30,32,35,36]. Multimodal rehabilitation approaches targeting both physical and psychological recovery, have positive effects on quality of life [5]. In line with these findings, both groups demonstrated improvements in physical and psychological status in the present study. Exercise is an indispensable therapeutic intervention for PCC management, as it is for many acute and chronic conditions. Many biological, psychological, and social factors play a role in the success of exercise, such as modulating the immune system, improving mitochondrial function, promoting neuroplasticity, increasing endorphin release, restoring the individual’s self-confidence, and facilitating social interaction [30,37,38].

We observed greater pain reduction in the VRG. The active, task-oriented, and interactive environment provided by VR exercises, with its multimodal and engaging stimuli, significantly engages and even challenges cognitive resources [39]. Within the framework of gate control theory, this diverts the brain’s attention away from painful stimuli, leading to a reduction in pain perception [39,40]. Furthermore, VR applications can also increase neuroplasticity and problem-solving ability by combining physical and cognitive tasks [40]. This suggests that the analgesic effect provided by VR exercises is not limited to distraction but may also involve cognitive processing through sensorimotor integration [41]. It is suggested that VR modifies the pain matrix in the central nervous system by altering the functional neural network and activating descending pain inhibition pathways, thereby directly affecting pain perception and processing [41,42].

Exercise is a recognized method for alleviating fatigue that arises in the PCC [5,30,31,32,33,35,38,43,44]. However, conventional exercise may be perceived by patients as an unpleasant obligation, leading to low motivation and poor compliance, also can exacerbate symptoms [45]. Furthermore, fatigue traps individuals in a vicious cycle, leading to a decrease in physical activity, a subsequent decline in fitness, and ultimately, intensified fatigue [39]. VR exercise, by contrast, may help break this obstacle by transforming exercise into a more engaging, motivating, and enjoyable experience, thereby freeing individuals from this cycle [43]. Evidence indicates that VR environments in PCC may increase compliance with training programs and enhance participant satisfaction [43]. It has also been reported that the gamified version of VR leads to physiological improvements that directly reduce fatigue, such as raising the fatigue threshold [18]. Ahmad et al. similarly found that VR exercise reduced fatigue but found no difference compared to conventional exercise [43]. That is likely due to their use of treadmill-based VR rather than motion-controlled video game–mediated exercise.

We found a significant time–group interaction in favor of VRG for depression scores, which aligns with previous studies reporting improvements in psychological distress following VR-based interventions during and after the COVID-19 pandemic [46,47]. The immersive environment, multimodal stimuli, and cognitive engagement offered by VR exercises may distance individuals from pessimistic mood states more effectively than traditional exercise by evoking a sense of presence and accomplishment, inducing positive emotions, facilitating behavioral activation, and reducing stress levels [17,46]. We observed a statistically significant decrease in anxiety levels among VRG participants, but this improvement did not differ significantly from that in the CTG. Considering that one of the critical factors in reducing anxiety levels is providing relaxation, this finding may suggest that the motion-controlled video game method we used did not provide a greater relaxation response than traditional exercise [48]. However, Rutkowski et al. also applied pulmonary rehabilitation enriched with a virtual therapeutic garden to provide relaxation in PCC patients, but reported no difference between groups despite a decrease in anxiety levels in both groups [16]. This finding may reflect the semi-immersive nature of our system, which may have limited the relaxation response.

Considering that aerobic bicycle ergometry was applied in the CTG, the comparable improvement in functional capacity observed in the VRG suggests that motion-controlled VR exercise may yield functional gains comparable to aerobic exercise programs. This possibility is supported by recent systematic reviews of chronic respiratory conditions, which demonstrate that VR- or video-game-based interventions produce exercise capacity improvements similar to traditional aerobic/pulmonary rehabilitation [10,49]. Similar findings have also been reached in a limited number of studies conducted in PCC [17,50].

The improvements in physical and psychological well-being induced by exercise were reflected in enhanced physical and mental quality-of-life scores and functional capacity, with post-treatment gains observed in both groups. However, the group effect favoring VR in pain, fatigue, and depressive symptoms did not translate into functional outcomes as expected. Symptom reductions are generally associated with increased mobility, exercise tolerance, and participation in daily activities, and the absence of such reflection in the present findings may be due to the relatively short intervention duration. The greater symptom-level benefits achieved through VR exercises may require a longer rehabilitation period to translate into functional and quality-of-life gains. In addition, functional improvements are influenced by multiple biopsychosocial factors, including physical conditioning, cognitive function, and social participation. Considering these multidimensional aspects of functionality, VR exercises may be more advantageous than traditional exercises.

These findings underscore the importance of integrating VR-based interventions within individual rehabilitation projects (IRPs), rather than applying them as isolated methods. An IRP represents a structured, person-centered rehabilitation plan that defines individualized goals, priorities, and interventions, along with the professionals responsible and the expected timeline for reassessment [51,52]. Within such a framework, motion-controlled VR exercise can serve as a flexible and adaptive component that supports personalized goal achievement through adjustable intensity, motivational engagement, and graded progression based on the patient’s evolving abilities and symptoms. Embedding VR exercise into the broader IRP structure can also facilitate interdisciplinary coordination (e.g., between physicians, physiotherapists, psychologists, and occupational therapists) and continuous feedback loops, ensuring that digital interventions remain aligned with the patient’s functional and psychosocial objectives. This approach may promote not only physical recovery but also autonomy, participation, and long-term self-management, which are key principles of modern rehabilitation medicine. Integrating such technology-driven modules into individualized rehabilitation projects may therefore enhance both the clinical effectiveness and the sustainability of rehabilitation in PCC.

There are several limitations to this study. First, it was conducted at a single center, which may limit the generalizability of the findings. Second, the sample size was determined based on effect sizes from a preliminary analysis and was powered for the primary outcome (VAS); therefore, it may not have been sufficient to detect smaller differences in some secondary outcomes. Third, our participants were individuals who had experienced mild-to-moderate acute COVID-19 that did not require hospitalization, which may restrict applicability to those with more severe disease or multisystem involvement. Fourth, there was no long-term follow-up to determine the persistence of the observed improvements or the sustainability of the benefits of VR exercise. Finally, participants were not assessed for PEM using a standardized instrument. However, we used FITT-VP principles to determine exercise intensity and routinely questioned participants regarding symptoms of PEM and other adverse effects before and after each session. No participants discontinued their sessions for reasons related to PCC symptoms or exercise intolerance.

Future studies should aim to replicate these findings in multi-center randomized controlled trials with larger and more heterogeneous samples to enhance generalizability. Long-term follow-up assessments are needed to determine the durability of improvements in pain, fatigue, and psychological outcomes and to explore whether these translate into meaningful functional and social gains. Additionally, cost-effectiveness analyses comparing VR exercise with standard physiotherapy and other engaging rehabilitation outlets, such as different exergaming platforms, immersive virtual environments, or tele-rehabilitation approaches, would provide valuable insight into the practical integration of such technologies into routine clinical care.

In conclusion, our findings suggest that, in individuals with mild to moderate PCC, motion-controlled video game–mediated VR exercises reduce pain and improve selected secondary outcomes under supervised conditions. Our findings also open the door to further exploring the potential of this innovative technology in unusual disease management.

## Figures and Tables

**Table 1:** Sociodemographic data of the participants.

	VRG (n = 37)	CTG (n = 37)	*p* Value
Age, years	43.3 (8.4)	45.3 (8.6)	0.30
Gender, female	30	28	0.77
BMI, kg/m^2^	26.4 (3.4)	27.2 (3.5)	0.31
Comorbidity, yes	10	8	0.81
Duration since COVID-19 diagnosis, months	9.1 (2.2)	8.7 (3.2)	0.67
Duration since PCC diagnosis, months	6.8 (1.3)	7.3 (1.7)	0.42
Quantitative variables are expressed as mean (standard deviation), categorical variables as n (%).			

**Table 2:** Baseline and post-intervention measurements of outcome measure.

	0. Week		8. Week		Change VRG	Change CTG
	VRG	CTG	VRG	CTG		
***Primary Outcome***						
**VAS**, *0*–*10* cm	6.4 (1.3)	6.1 (1.2)	4.2 (1.1)	5.3 (1.2)	−2.22 ± 0.95	−0.85 ± 0.57
***Secondary Outcomes***						
**FSS**	46.9 (8.2)	45.2 (7.1)	39.6 (9.1)	39.7 (7.8)	−8.16 ± 6.70	−3.97 ± 4.61
**HADS-A**	8.6 (2.2)	7.9 (3.2)	7.6 (2.1)	7.1 (2.8)	−1.03 ± 0.99	−1.19 ± 1.60
**HADS-D**	9.1 (3.0)	8.7 (3.1)	7.1 (2.1)	7.8 (2.5)	−2.00 ± 2.30	−0.84 ± 1.21
**6MWT**, meters	461.6 (88.8)	450.6 (44.5)	499.3 (74.1)	478.8 (42.1)	37.7 ± 40.3	27.8 ± 17.1
**SF-12 M**	42.4 (11.6)	44.8 (11.9)	48.7 (10.3)	53.2 (7.8)	6.23 ± 9.91	8.33 ± 10.84
**SF-12 F**	34.0 (9.4)	30.8 (9.4)	40.9 (7.0)	37.9 (9.9)	6.23 ± 9.91	7.04 ± 8.91

6MWT, Six Minute Walk Test; FSS, Fatigue Severity Scale; HADS-A/HADS-D, Hospital Anxiety and Depression Scale; VAS, Visual Analog Scale; SF-12 M/P, Short Form-12 Mental/Physical.

**Table 3:** Linear Mixed-Effects Model Results for Primary and Secondary Outcomes.

	Group			Time			Time × Group Interaction			
Outcome	Mean Diff [95% CI]	F (df_1_, df_2_)	*p*	Mean Diff [95% CI]	F (df_1_, df_2_)	*p*	Mean Diff [95% CI]	F (df_1_, df_2_)	*p*	Partial η^2^
***Primary Outcome***										
**VAS**, *0*–*10* cm	−0.26 [−0.84–0.32]	0.74 (1, 59.4)	0.387	−2.22 [−2.50–−1.93]	232.3 (1, 59.4)	**<0.001**	−1.37 [−1.73–−1.00]	56.31 (1, 59.4)	**<0.001**	0.487
***Secondary Outcomes***										
**FSS**	+1.73 [−1.38–4.84]	1.21 (1, 61.5)	0.275	−6.07 [−7.11–−5.03]	93.8 (1, 61.5)	**<0.001**	−4.19 [−6.99–−1.39]	8.96 (1, 61.5)	**0.004**	0.127
**HADS-A**	+0.73 [−0.67–2.13]	1.10 (1, 60.0)	0.298	−1.11 [−1.62–−0.60]	17.3 (1, 60.0)	**<0.001**	+0.16 [−0.45–0.78]	0.28 (1, 60.0)	0.601	0.005
**HADS-D**	+0.32 [−1.16–1.80]	0.17 (1, 54.6)	0.680	−1.42 [−1.92–−0.92]	34.9 (1, 54.6)	**<0.001**	−1.16 [−2.02–−0.31]	7.40 (1, 54.6)	**0.009**	0.119
**6MWT**, meters	+11.0 [−12.1–34.1]	0.92 (1, 48.8)	0.340	+32.7 [19.8–45.6]	45.6 (1, 48.8)	**<0.001**	+9.9 [−4.6–24.4]	1.90 (1, 48.8)	0.175	0.037
**SF-12 Physical**	+3.16 [−1.01–7.33]	2.28 (1, 69.5)	0.136	+6.95 [5.25–8.65]	74.8 (1, 69.5)	**<0.001**	−0.18 [−3.97–3.61]	0.009 (1, 69.5)	0.926	0.0001
**SF-12 Mental**	−2.39 [−7.33–2.55]	0.92 (1, 71.4)	0.340	+7.53 [5.12–9.94]	41.2 (1, 71.4)	**<0.001**	−2.10 [−6.91–2.72]	0.76 (1, 71.4)	0.388	0.010

6MWT, Six Minute Walk Test; FSS, Fatigue Severity Scale; HADS-A/HADS-D, Hospital Anxiety and Depression Scale; VAS, Visual Analog Scale; SF-12 M/P, Short Form-12 Mental/Physical.

## Data Availability

The data presented in this study are available on request from the corresponding author. The data are not publicly available due to privacy and ethical restrictions.
